# Unlocking the Real Potential of Black Soldier Fly (*Hermetia illucens*) Larvae Protein Derivatives in Pet Diets

**DOI:** 10.3390/molecules26144216

**Published:** 2021-07-11

**Authors:** Ange Mouithys-Mickalad, Nuria Martin Tome, Thomas Boogaard, Arpita Chakraborty, Didier Serteyn, Kees Aarts, Aman Paul

**Affiliations:** 1 Centre of Oxygen, Research and Development, University of Liege, 4000 Liege, Belgium; amouithys@uliege.be (A.M.-M.); nto@protix.eu (D.S.); 2 Protix B.V., 5107 NC Dongen, The Netherlands; didier.serteyn@uliege.be (N.M.T.); arpita.chakraborty@protix.eu (A.C.); kees.aarts@protix.eu (K.A.); 3 Nutrilab B.V., 4283 GG Giessen, The Netherlands; t.boogaard@nutrilab.nl

**Keywords:** *Hermetia illucens*, chicken meal, arthritis, pets, proteinase, erythrocytes, macrophages, monocytes, glucosamine

## Abstract

Black soldier fly larvae (BSFL)-derived proteins are gaining popularity as sustainable pet food ingredients. According to the literature, these ingredients have strong antioxidant and antimicrobial activities. Due to the ability of BSFL protein derivatives to donate hydrogen atoms and/or electrons to counterpoise unstable molecules, they could possibly help in the prevention of osteoarthritis. During this study, the antiarthritic potential of BSFL protein derivatives was evaluated using the following assays: (1) proteinase inhibition, (2) erythrocyte membrane stability, (3) reactive oxygen species (ROS) production by activated macrophages, (4) ROS production by monocytes, and (5) cellular toxicity. Additionally, the glucosamine content of these ingredients was also evaluated. Chicken meal is commonly used in pet food formulations and was used as an industrial benchmark. The results obtained during this study demonstrated the strong antiarthritic potential of BSFL protein derivatives. We found that BSFL protein derivatives are not only useful in preventing the development of arthritis but could also help to cure it due to the presence of glucosamine. We also found that chicken meal could contribute to the development of arthritis by increasing ROS production by monocytes.

## 1. Introduction

Black soldier fly larvae (BSFL)-derived proteins are becoming increasingly popular to help reduce the carbon footprints of pet food formulations [1,2,3]. These ingredients are nutritious, highly digestible and could promote the health of animals that consume them [4,5,6]. Currently, pet food is the biggest market for insect proteins in Europe [7]. Globally, 50% of households own a cat or a dog. Together, these two companion animals are responsible for 95% of global pet food sales [8]. The health and wellbeing of these companion animals are of prime importance to their owners [9].

Osteoarthritis (OA) is one of the common pathologies affecting dogs and cats. It may affect the knee, hip, shoulder, and/or elbow of the animal [10]. OA initiation and progression is associated with several factors. Ageing-related stress could result in the secretion of molecules such as chemokines, cytokines, and proteases. These molecules either themselves react, or trigger the secretion of compounds that react with cellular components, leading to degradation or structural changes in the synovial membrane, subchondral bone, and/or articular cartilage. As a result of these processes, pets may develop impaired joint functioning as well as painful locomotion [11,12]. Curative treatment of OA involves the use of nonsteroidal anti-inflammatory drugs (NSAIDs), such as acetaminophen, which are often associated with several side effects (e.g., gastrointestinal problems) [13]. Veterinary professionals emphasize the preventive approach by encouraging the use of natural pet food ingredients that could proactively prevent the development of OA [10]. In our previous publication, we demonstrated the ability of BSFL protein derivatives to protect animal cells against neutrophil-mediated oxidative damage. Due to the ability of BSFL protein derivatives to donate hydrogen atoms and/or electrons to counterpoise unstable molecules [14], they could possibly help in the prevention of OA formation. However, to the best of our knowledge, there are currently no publications that evaluate the potential role of BSFL protein derivatives in OA prevention.

During this study, we conducted multiple in vitro assays to investigate the preventive role of BSFL protein derivatives in various pathways leading to (osteo) arthritis formation. This is the first study to evaluate the antiarthritic potential of insect proteins. Chicken meal is commonly used in pet food formulations as a protein source and hence was used as an industrial benchmark during this study.

## 2. Results

### 2.1. Glucosamine Content

The glucosamine content of BSFL protein derivatives and chicken meal is indicated in Table 1. There were no significant differences in the glucosamine content amongst the tested samples (*p* < 0.05).

### 2.2. Proteinase (Trysin) Inhibitory Assay

**Figure 1:**
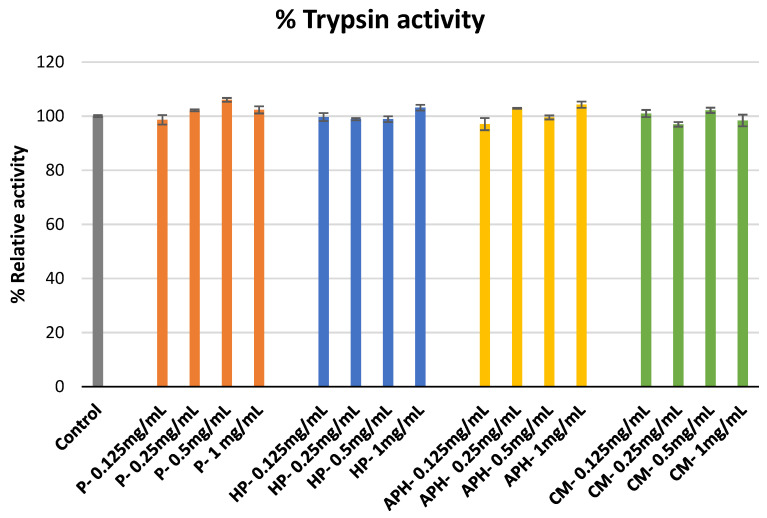
Modulation of trypsin catalyzed azocasein hydrolysis by P (water-soluble extract of pasteurized minced meat of BSFL), HP (water-soluble extract of hydrolyzed and pasteurized minced meat of BSFL), APH (hydrolysate of water-soluble BSFL proteins), and CM (water-soluble extract of chicken meal). Results expressed as mean ± standard deviation (n = 3).

The percentage inhibition of trypsin activity by hydrolysate of water-soluble BSFL protein (APH), pasteurized minced meat of BSFL water soluble extract (P), hydrolyzed and pasteurized minced meat of BSFL water soluble extract (HP), and chicken meal water soluble extract (CM) is indicated in Figure 1. None of the samples tested during this study demonstrated trypsin inhibition activity.

### 2.3. Erythrocyte Membrane Stability Assay

**Figure 2:**
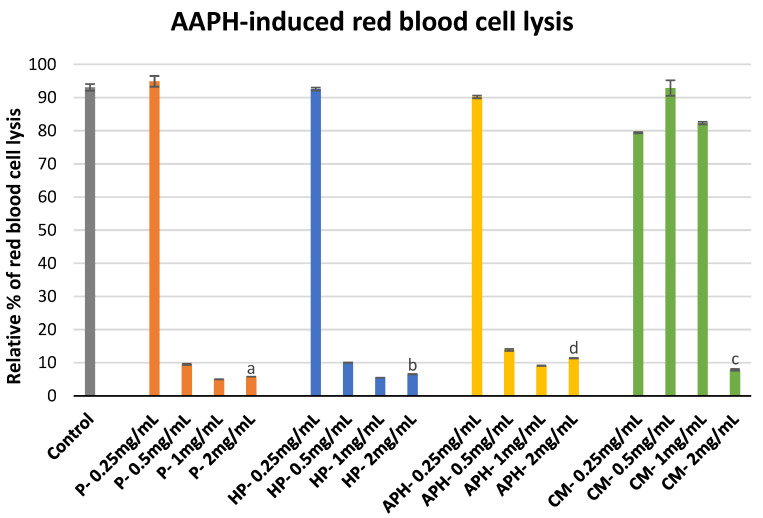
Modulation of AAPH -induced red blood cell lysis by P (water-soluble extract of pasteurized minced meat of BSFL), HP (water-soluble extract of hydrolyzed and pasteurized minced meat of BSFL), APH (hydrolysate of water-soluble BSFL proteins), and CM (water-soluble extract of chicken meal). Results expressed as mean ± standard deviation (n = 3). Differences in the letters above the bars (a, b, c, and d) represent significant differences (*p* < 0.05) in prevention of AAPH-induced red blood cell lysis by different samples analyzed during this assay.

The protective effect of P, HP, APH, and CM against free radical-induced red blood cell (erythrocyte) lysis is indicated in Figure 2. During this assay P, HP, and APH initially showed an increase in hemolysis inhibition when concentration was increased from 0.25 to 1 mg/mL. Afterward, there was a slight decrease in inhibition with an increase in concentration from 1 to 2 mg/mL. Whereas for CM there was no considerable increase in hemolysis inhibition at first, with an increasing concentration from 0.25 to 1 mg/mL, finally, at the maximum concentration tested (2 mg/mL), CM demonstrated considerable inhibition activity. At the highest concentration used, the percentage inhibition was in the following order: P > HP > CM > APH (*p* < 0.05). These results indicate that pasteurized meat of BSFL (P) is the most effective in protecting the red blood cells against AAPH (2,2′-Azobis(2-methylpropionamidine) dihydrochloride)-induced cell lysis amongst all the tested samples. The significant differences in free radical induced red blood cell lysis by different samples at highest concentration tested (2 mg/mL) are indicated in Figure 2 with letters.

### 2.4. Reactive Oxygen Species (ROS) Production by Macrophages

**Figure 3:**
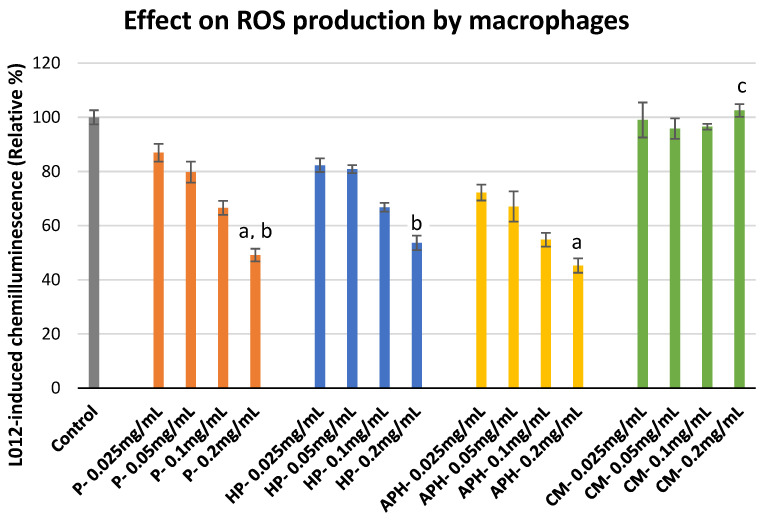
ROS production modulation in PMA (phorbol 12-myristate 13-acetate)-activated macrophages after exposure to P (water-soluble extract of pasteurized minced meat of BSFL), HP (water-soluble extract of hydrolyzed and pasteurized minced meat of BSFL), APH (hydrolysate of water-soluble BSFL proteins), and CM (water-soluble extract of chicken meal). Results expressed as mean ± standard deviation (n = 3). Differences in the letters above the bars (a, b, and c) represent significant differences (*p* < 0.05) in prevention of ROS production (via macrophages) by different samples analyzed during this assay.

The effect of P, HP, APH and CM on the ROS production by macrophages is indicated in Figure 3. For P, HP and APH there was a decrease in chemiluminescence response alongside an increase in concentration (i.e., decreased ROS production). In contrast, for CM there was no change in chemiluminescence at any of the tested concentrations. This indicated that all the BSFL protein derivatives suppressed ROS production, whereas CM had no effect on the macrophages’ ROS production. Significant differences in the inhibition of ROS production by different samples at highest concentration tested (0.2 mg/mL) are indicated with letters in Figure 3.

### 2.5. ROS Production by PMA Activated HL-60 Cells

**Figure 4:**
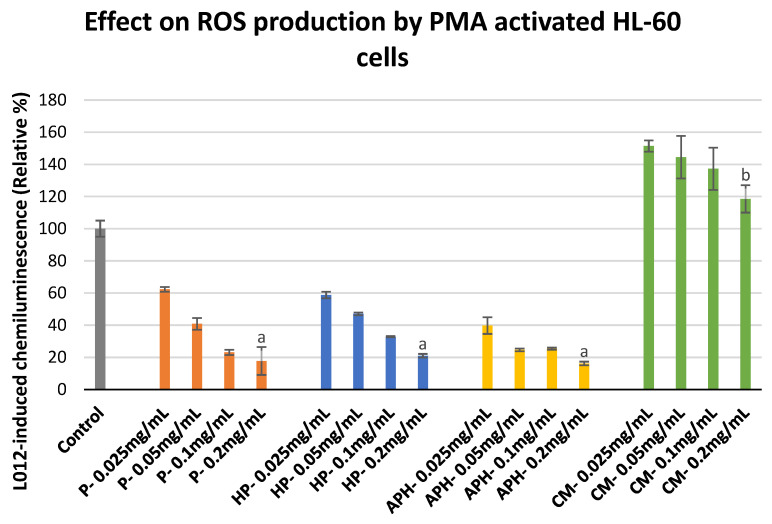
ROS production modulation in PMA-activated HL-60 cells after exposure to P (water-soluble extract of pasteurized minced meat of BSFL), HP (water-soluble extract of hydrolyzed and pasteurized minced meat of BSFL), APH (hydrolysate of water-soluble BSFL proteins), and CM (water-soluble extract of chicken meal). Results expressed as mean ± standard deviation (n = 3). Differences in the letters above the bars (a and b) represent significant differences (*p* < 0.05) in prevention of ROS production (via PMA activated HL-60 cells) by different samples analyzed during this assay.

Figure 4 shows the effect of increasing the concentration of P, HP, APH, and CM on the ROS production of PMA-activated HL-60 cells. A decrease in ROS production was observed with increasing concentrations of P, HP, and APH. In the case of CM, at the lowest concentration tested (0.025 mg/mL), ROS production was 1.5-fold higher in comparison to the control (activated macrophages alone taken as 100%). The ROS production decreased with increasing concentrations of insect samples. However, even at the highest concentration tested (0.2 mg/mL), CM exhibited a rather pro-inflammatory behavior (i.e., promoting ROS production). At the highest tested concentration (0.2 mg/mL), the percentage relative chemiluminescence was in the following order: CM > P = HP = APH. The significant differences in the inhibition of ROS production in activated monocytes by different samples at highest concentration tested (0.2 mg/mL) are also indicated in Figure 4 with letters.

### 2.6. Metabolic Activity of HL-60 Cells

**Figure 5:**
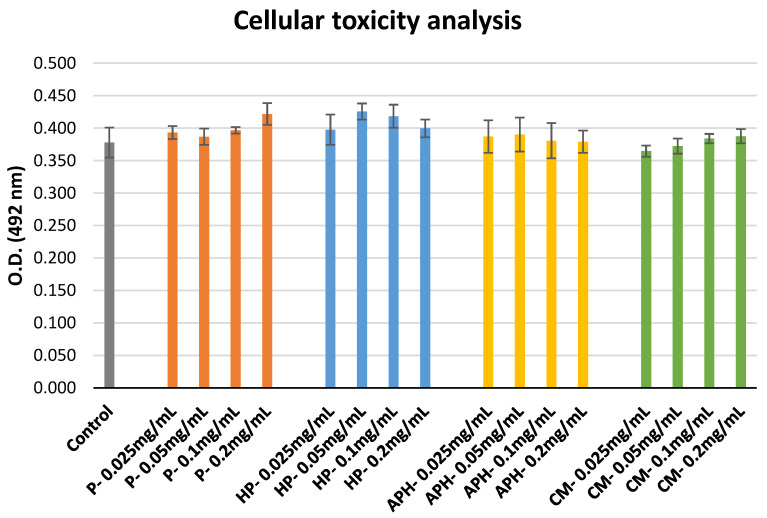
Cellular toxicity of P (water-soluble extract of pasteurized minced meat of BSFL), HP (water-soluble extract of hydrolyzed and pasteurized minced meat of BSFL), APH (hydrolysate of water-soluble BSFL proteins), and CM (water-soluble extract of chicken meal). Results expressed as mean ± standard deviation (n = 3).

The outcomes of the cellular toxicity analysis are indicated in Figure 5. None of the samples exhibited toxicity, irrespective of the concentration tested in comparison to the control (cells alone + MTS).

## 3. Discussion

### 3.1. Glucosamine Content

Glucosamine and its salts are commonly used as nutraceutical supplements to ease the pain in dogs suffering from OA. As an amino monosaccharide, glucosamine is the preferred substrate for the biosynthesis of glycosaminoglycan, which is further used for the biosynthesis of proteoglycans that form the extracellular matrix and make up the cartilage [15]. Three insect protein samples tested during this study contained 0.4 to 0.5% glucosamine (on a dry matter basis) in the monomeric form. Therefore, a dry pet food formulation containing 30% of these insect proteins can supply approximately 120 mg glucosamine per 100 g of dry pet food formula. A popular pet food brand currently recommends feeding 250 to 340 g of insect-based formula per day to dogs with a body weight of 20 to 30 kg [16]. This feeding pattern will render about 300 to 400 mg glucosamine to the dog consuming it, which is a considerable amount when compared to commercial glucosamine supplements available on the market that contain about 300 to 1600 mg glucosamine [17]. However, there are some reports that provide evidence of the limited uptake of orally administered glucosamine in dogs [18]. In future, it could be of interest to evaluate the in vivo uptake and kinetics of glucosamine present in BSFL proteins when included in pet food formulations.

### 3.2. Proteinase Inhibition

The development of OA occurs in three distinct phases: (a) phase 1—proteinase-mediated hydrolysis of cartilage matrix; (b) phase 2—fibrillation and disintegration of the cartilage surface that is coupled with the release of disintegration products into synovial fluid; and (3) phase 3—the engulfing of disintegration products by synovial cells (via phagocytosis) and production of proteinase (participating in the phase 1 reaction) and cytokines that initiate inflammation [19]. Metalloproteases and serine proteinase are considered to have a key role in the hydrolysis of cartilage matrix [20]. During this study, we used trypsin, which is a serine proteinase, to evaluate the proteinase inhibition activity of BSFL protein derivatives and chicken meal. None of the tested samples were able to inhibit the trypsin activity (see Figure 1), indicating that BSFL protein derivatives would not have any role in preventing OA during phase 1.

It is also important to note the other consequences of these results. Trypsin is an important proteinase secreted in the digestive tracts of several vertebrates [21]. Plant proteins (including soy meal) contain different types of trypsin inhibitors, which irreversibly bind with trypsinogen to reduce the digestibility of proteins [22]. Interestingly, here, we provide evidence that the BSF protein derivatives used in the study did not inhibit trypsin, indicating their nutritional superiority against some plant proteins.

### 3.3. Erythrocyte Membrane Stability

Erythrocyte stability is crucial during rheumatoid arthritis. The literature published during the last decade has also highlighted the importance of erythrocytes in OA. There is already evidence to indicate an increase in erythrocyte sedimentation rate during OA [23]. The increase in erythrocyte sedimentation rate could be attributed to the assault of ROS [24] produced during the third phase of the OA process. Furthermore, if the concentration of viable erythrocytes in the joint cavity decreases, it can have severe implications for the cartilage and synovial tissues [25]. During this assay, we used AAPH to generate ROS. AAPH is commonly used in biochemical assays to investigate the cytoprotective effects of amino residues [26]. AAPH can generate free radicals via spontaneous decomposition (at body temperature, i.e., 37 °C). These free radicals can react with oxygen to produce ROS, which can further react with lipids present in the cellular membrane to form peroxyl radicals. This process not only results in the production of peroxyl radicals that participate in inflammatory process, but also results in the disintegration of cells (including erythrocytes) [26]. Molecules that can stabilize the reactive products generated by AAPH are known to have cytoprotective effects [27] and could contribute towards the prevention of OA.

During this study, BSFL protein derivatives demonstrated strong cytoprotective activity at all the concentrations ≥ 0.5 mg/mL (see Figure 2). In our previous study, we demonstrated the strong potential of BSFL protein derivatives to donate hydrogen atoms and electrons in stabilizing free radicals [14]. This suggests that BSFL protein derivatives could instantly donate these chemical species to stabilize the intermediates of AAPH-induced oxidation resulting in cytoprotective effects in dogs. At the highest concentration, out of all the tested samples, P (pasteurized minced meat of insects) exhibited the maximum inhibition of cell lysis.

At concentrations up to 1 mg/mL, CM showed little to no cytoprotective activity. Surprisingly, at 2 mg/mL, CM showed strong cytoprotective activity. Amino acids, such as cysteine, tryptophan, histidine, and tyrosine, are known to effectively protect the cells against AAPH assault [28]. Depending on their chemical state (free amino acid, di-, tripeptide, etc.), some of these amino acids have different rates of consumption during AAPH assault. For example, at low concentrations, di- and tri-tryptophan are consumed twice when compared to free tryptophan to participate in AAPH-induced oxidation. However, at higher concentrations, all the forms of tryptophan have similar rates of consumption [26]. Therefore, it is possible that in the case of CM, some active amino acids are present in a chemical form that are more active at higher concentrations.

### 3.4. ROS Production by Macrophages

Macrophages play a key role in animals’ bodies due to their ability to identify, engulf, and destruct pathogenic microbes or substances during phagocytosis. During OA, the activated macrophages express NOX2 genes that trigger the secretion of NADH oxidase, resulting in the production of ROS including superoxide anions [29]. These ROS are further responsible for chondrocyte senescence and cartilage breakdown [29,30]. During this study, we found that all three BSFL protein derivatives were able to suppress ROS production by macrophages. At the highest concentration used, P and APH were able to suppress ROS production > 50% in comparison to the control, whereas no ROS suppression activity was observed in the case of CM. ROS suppression activity could possibly arise due to two mechanisms: (a) The scavenging of ROS produced by macrophages. In our previous study, we already demonstrated the strong potential of BSFL protein derivatives to scavenge ROS. We also demonstrated that CM was ineffective in scavenging ROS [14]. (b) The downregulation of NOX2 genes. There is well-documented evidence regarding the ability of specific food-derived bioactive peptides to downregulate genes responsible for ROS production [31]. It is possible that BSFL protein derivatives also have specific peptides that could result in such downregulation. Results indicated in this section show that BSFL protein derivatives can suppress ROS production from macrophages and could possibly help in the prevention of OA in pets. Furthermore, it could be interesting to conduct future studies for evaluating the ability of BSFL peptides to downregulate the genes responsible for inflammation.

### 3.5. ROS Production by HL-60 Cells

Oxidative stress could trigger ROS production in monocytes [29], which may further contribute to the development of OA. We used PMA-activated HL-60 cells to mimic monocytes. During this study, we found that BSFL protein derivatives were highly effective in suppressing the ROS production by HL-60 cells. We believe the ROS suppression in this case could again be explained by the mechanisms listed in Section 3.4. Whereas CM exhibited pro-inflammatory activity at all tested concentrations, a downward trend (decreasing pro-inflammatory activity) was observed for CM with an increase in concentration.

### 3.6. Cellular Metabolic Activity

None of the tested samples had a negative impact on the viability of HL-60 cells during this assay. This provides the following indication: (a) BSFL protein derivatives are not toxic to animal cells at all tested concentrations; (b) the ROS suppression activity seen in previous sections did not cause as a result of cellular mortality.

Free amino acids, short chain peptides, and water-soluble proteins could pass the intestinal membrane intact with minimum alterations as a result of digestion. This indicates that water-soluble extracts could be similarly in the animal body without the interference of the digestive process and thus, similar activities as the ones reported in this study can be expected in vivo. Additionally, APH is highly water-soluble (> 95%) and contains 100% proteins < 1000 Da (i.e., short chain peptides) [14], which makes it an interesting candidate for arthritis prevention. Furthermore, it will be interesting to validate the results of this in vitro study with animal feeding trials.

## 4. Materials and Methods

### 4.1. Reagents

All the reagents were of analytical grade. Dimethylsulfoxide (DMSO), ethanol, CaCl_2_, NaCl, and hydrogen peroxide (H_2_O_2_) were supplied by Merck (VWRI, Leuven, Belgium). Sodium nitrite (NaNO_2_), bovine serum albumin (BSA), PMA, trypsin (CAS number 102110-74-7), and azocasein (CAS number 9002-07-7) were purchased from Sigma-Aldrich (Bornem, Belgium). 8-Amino-5-chloro-7-phenylpyrido [3,4-d] pyridazine-1,4 (2H,3H) dione (L-012) was purchased from Wako Chemicals (Neuss, Germany). MTS Cell Titer 96R was purchased from Promega REF G5430 (Madison, USA). All aqueous solutions were prepared with water previously purified using a Milli-Q water system (Millipore, Bedford, MA, USA). Stock solutions of BSFL protein derivative and chicken meal were prepared at concentrations of 10 or 20 mg/mL. The final concentrations were obtained by successive dilutions with distilled Milli-Q.

### 4.2. Raw Materials

Chicken meal was purchased from an online shop in October 2020. Three types of BSFL protein derivatives, namely, pasteurized minced BSFL meat, enzymatically hydrolyzed and pasteurized minced BSFL meat, and hydrolysate of water-soluble BSFL proteins (APH), were supplied by Protix B.V. (Dongen, The Netherlands) in November 2020. Product composition (see Table 2 for proximate composition), storage conditions, and the method employed to develop the water-soluble extract were similar to those detailed in our previous publication [14]. Glucosamine content was measured in BSFL protein derivatives as well as chicken meal. For biochemical investigations, APH was used as it is, due to its high water solubility. However, in the case of other raw materials (the other two BSFL protein derivatives and chicken meal), due to limited water solubility, respective water-soluble extracts (P, HP, and CM) were used for testing.

Human myeloid HL-60 cell line was purchased from American Type Culture Collection (Manassas, USA) and cultured according to the method described by Boly et al. [32]. At the beginning of each assay, (1) the cell count of the suspension was estimated to maintain cellular density of 10^6^ cells/mL and (2) cell viability was measured using trypan blue assay to main viability > 95%.

Testing concentrations for Section 4.4, Section 4.5, Section 4.6, and Section 4.7 were selected based on preliminary experimentations to ensure that the minimal tested concentration yielded absorbance or chemiluminescence of no more or less than 50% of the values obtained for control in each assay.

### 4.3. Glucosamine Content

Glucosamine content was analyzed by Eurofins Food Testing B.V. (Barendrecht, The Netherlands). BSFL protein derivatives and chicken meal samples were mixed with phenyl isothiocyanate in a pre-column derivatization reaction. Then, these samples were separated on a reverse phase ultra-liquid chromatography system equipped with an ethylene bridged hybrid column. The quantification of peaks was performed against an external standard [33].

### 4.4. Proteinase Inhibitory Assay

The proteinase inhibition abilities of P, HP, APH, and CM were evaluated using the protocol of Murugesan et al. [34]. Reaction mixtures were obtained by mixing 1 mL of 25 mM tris-HCl buffer and 0.06 mg trypsin with 1 mL sample solution (at 0.125, 0.25, 0.5 and 1 mg/mL). This was followed by incubation for 10 min at 37 °C, the addition of 1 mL azocasein solution (0.8%), and a final incubation for 20 min. Then, 2 mL of perchloric acid (70%) was added to the mixtures to arrest the reaction. The resulting mixtures were centrifuged, and the absorbance of respective supernatants was measured at 450 nm wavelength. Inhibition activities (%) were calculated using the following formula: (absorbance of control—absorbance of respective sample)/(absorbance of control) × 100.

### 4.5. Erythrocyte Membrane Stability Assay

Cell membrane stability assay was realized according to the protocol of Karimi et al. [27]. The blood used for the assay was collected from a healthy horse in 10 mL tubes containing EDTA (Ethylenediaminetetraacetic acid). It was centrifuged for 10 min at 3000 rpm and plasma supernatant was discarded. The resulting erythrocytes were washed twice with 5 mL PBS solution at pH 7.4. Red blood cell pellets were diluted 10 times by phosphate buffer solution (PBS). Aliquots (0.5 mL) of this cell suspension were transferred into a 5 mL tube. This was followed by the addition of 0.35 mL of 0.15 M phosphate buffer, 0.1 mL sample solution (at 0.25, 0.5, 1 and 2 mg/mL), and incubation for 10 min. The red blood cell lysis was triggered by the addition of 0.05 mL 25 mM 2,2′-Azobis(2-methylpropionamidine) dihydrochloride (AAPH). For the control, only a buffer solution was used instead of water-soluble extract solutions. The resulting mixtures were incubated for 30 min at 57 °C. Finally, the mixtures were allowed to stand at room temperature and centrifuged for 10 min at 3000 rpm. The supernatants (0.5 mL) were diluted with PBS, and the absorbance was measured at 560 nm. The percentage of inhibition toward red blood cell lysis from AAPH was estimated using the formula mentioned in Section 4.4.

### 4.6. Cellular ROS Production

#### 4.6.1. ROS Production by Macrophages

Cultured HL-60 cells were plated (1 × 10^6^ cells/mL in 24-well plate) in Iscove’s Modified Dulbecco’s Medium. The differentiation of monocytes was induced by adding 10 nM PMA dissolved in DMSO and incubation for 24 h at 37 °C. It was ensured that the final concentration of DMSO in the culture medium was <0.1 % and that DMSO addition did not impact HL-60 proliferation, viability, or differentiation. The morphological changes (to macrophage phenotype) in HL-60 cells resulting from differentiation were verified after 24 h of culturing using light microscopy. Post-differentiation, the medium was discarded, and nonadherent cells were gently washed with Hank’s balanced salt solution (HBSS). Only the adherent cells were used for the assay.

ROS produced by macrophages (or HL-60 cells) were estimated by chemiluminescence (CL) measurement. During this assay, L-012 was used as a CL enhancer using a method adapted from Lelciu et al. [35]. Macrophages were treated with 0.05 mL of sample solution to reach final concentrations of 0.025, 0.05, 0.1, and 0.2 mg/mL and incubated for 1 h in the presence of 0.8 mL HBSS. Then, 20 µL of Ca^2+^ (10 mM) and 20 µL of L-012 (10^−4^ M) were added prior to the activation with 0.05 mL PMA to reach the final volume of 1 mL. The CL response of macrophages was monitored for 30 min at 37 °C using a Fluoroskan Ascent (Fisher Scientific, Tournai, Belgium) and expressed as the integral value of total CL emission. For the control only, HBSS was added instead of water-soluble extract solution.

#### 4.6.2. ROS Production by PMA Activated HL-60 Cells

Cultured HL-60 (5 × 10^5^ cells) were suspended in 143 µL HBSS and loaded in each well of a 96-well microtiter plate. They were incubated at 37 °C for 10 min with 2 µL of sample solution to reach final concentrations of 0.025, 0.05, 0.1, and 0.2 mg/mL. Post-incubation, 20 µL of Ca^2+^ (10 mM) and 20 µL of L-012 (10^−4^ M) were added into each well. Finally, the mixtures were activated with 10 µL PMA (16 µM), and the CL was measured for 30 min at 37 °C using a Fluoroskan Ascent (Fisher Scientific, Tournai, Belgium). Control assays were realized as indicated in Section 4.5. The CL response was expressed as an integral value of total CL emission.

### 4.7. Metabolic Acitivty of HL-60 Cells

Cultured HL-60 cells (1 × 10^4^ cells/well) were incubated with 0.025, 0.05, 0.1, and 0.2 mg/mL of each sample solution for 1 h at 37 °C. Post-incubation, treated cells were washed twice with media, and the cell metabolic activity was evaluated by adding 10 µL MTS tetrazolium salt as a cytotoxicity indicator. The absorbance of the mixtures was measured every 60 min for 2 h.

### 4.8. Statistical Analysis

All the testing during this study was performed in triplicates. Significant differences in the values obtained during glucosamine, cell membrane stability, and ROS production by macrophages, as well as PMA activated HL-60 cell analyses, were examined using one-way ANOVA. Subsequently, Tukey’s Range Test was performed to identify which differences were statistically significant. Differences between means were considered significantly different if the *p*-value was less than 0.05. This analysis was conducted in GraphPad Prism 8 (GraphPad Software, San Diego, CA, USA).

## 5. Conclusions

We demonstrated the strong antiarthritic potential of BSFL protein derivatives during this study. We found that BSFL protein derivatives were not only useful in preventing the formation of arthritis (red blood cell stability and ROS production reduction in macrophages and monocytes) but could also help in curing it due to the presence of glucosamine. During this study, we found that chicken meal, which is commonly used in pet food formulations, could contribute to arthritis development by increasing the ROS production by monocytes.

## Figures and Tables

**Table 1:** Glucosamine content of BSFL protein derivatives and chicken meal.

Sample	Glucosamine Content (%)
Pasteurized minced meat of BSFL	0.5 ± 0.2 ^a^
Hydrolyzed and pasteurized minced meat of BSFL	0.5 ± 0.2 ^a^
Hydrolysate of water-soluble BSFL proteins	0.4 ± 0.0 ^a^
Chicken meal	0.4 ± 0.0 ^a^

Data are presented as mean ± standard deviation (n = 3). Differences in the superscript letters represent significant differences (*p* < 0.05) in the glucosamine content of different samples.

**Table 2:** Proximate composition of BSFL protein derivatives and chicken meal as indicated by supplier (as in basis).

Sample	Moisture Content (g/kg)	Crude Protein Content (g/kg)	Crude Fat Content (g/kg)
Pasteurized minced meat of BSFL	700.0	120.0	122.5
Hydrolyzed and pasteurized minced meat of BSFL	700.0	120.0	122.5
Hydrolysate of water-soluble BSFL proteins	55.0	455.0	35.0
Chicken meal	60.0	700.0	120.0

## Data Availability

The data presented in this study are available on request from the corresponding author. The data are not publicly available due to patent protection.

## Sample Availability

Samples of the compounds pasteurized minced meat of BSFL water soluble extract (P), hydrolyzed and pasteurized minced meat of BSFL water soluble extract (HP), hydrolysate of water-soluble BSFL protein (APH), and chicken meal water soluble extract (CM) are available from the authors.

## References

[1] Acuff H.L., Dainton A.N., Dhakal J., Kiprotich S., Aldrich G. (2021). Sustainability and Pet Food: Is There a Role for Veterinarians?. Vet. Clin. N. Am. Small Anim. Pract..

[2] Smetana S., Schmitt E., Mathys A. (2019). Sustainable Use of *Hermetia Illucens* Insect Biomass for Feed and Food: Attributional and Consequential Life Cycle Assessment. Resour. Conserv. Recycl..

[3] Terrey D., James J., Tankovski I., Dalim M., van Spankeren M., Chakraborty A., Schmitt E., Paul A. (2021). Palatability Enhancement Potential of *Hermetia Illucens* Larvae Protein Hydrolysate in *Litopenaeus vannamei* Diets. Molecules.

[4] Freel T.A., McComb A., Koutsos E.A. (2021). Digestibility and Safety of Dry Black Soldier Fly Larvae Meal and Black Soldier Fly Larvae Oil in Dogs. J. Anim. Sci..

[5] Dong L., Ariëns R.M.C., America A.H.P., Paul A., Veldkamp T., Mes J.J., Wichers H.J., Govers C. (2021). *Clostridium perfringens* Suppressing Activity in Black Soldier Fly Protein Preparations. LWT.

[6] Veldkamp T., Dong L., Paul A., Govers C. (2021). Bioactive Properties of Insect Products for Monogastric Animals—A Review. J. Insects Food Feed.

[7] International Platform of Insect for Food and Feed (IPIFF) IPIFF Publications. https://ipiff.org/wp-content/uploads/2020/05/IPIFF-RegulatoryBrochure-update07-2020-1.pdf.

[8] Alexander P., Berri A., Moran D., Reay D., Rounsevell M.D.A. (2020). The Global Environmental Paw Print of Pet Food. Glob. Environ. Chang..

[9] Chan M.M., Tapia Rico G. (2019). The “Pet Effect” in Cancer Patients: Risks and Benefits of Human-Pet Interaction. Crit. Rev. Oncol. Hematol..

[10] Comblain F., Serisier S., Barthelemy N., Balligand M., Henrotin Y. (2016). Review of Dietary Supplements for the Management of Osteoarthritis in Dogs in Studies from 2004 to 2014. J. Vet. Pharmacol. Ther..

[11] Buckwalter J.A., Lotz M.K., Stoltz J.F. (2007). Osteoarthritis, Inflammation and Degradation: A Continuum (Vol. 70).

[12] Coryell P.R., Diekman B.O., Loeser R.F. (2021). Mechanisms and Therapeutic Implications of Cellular Senescence in Osteoarthritis. Nat. Rev. Rheumatol..

[13] Johnston S.A., McLaughlin R.M., Budsberg S.C. (2008). Nonsurgical Management of Osteoarthritis in Dogs. Vet. Clin. N. Am. Small Anim. Pract..

[14] Mouithys-Mickalad A., Schmitt E., Dalim M., Franck T., Tome N.M., van Spankeren M., Serteyn D., Paul A. (2020). Black Soldier Fly (*Hermetia Illucens*) Larvae Protein Derivatives: Potential to Promote Animal Health. Animals.

[15] D’Altilio M., Peal A., Alvey M., Simms C., Curtsinger A., Gupta R.C., Canerdy T.D., Goad J.T., Bagchi M., Bagchi D. (2007). Therapeutic Efficacy and Safety of Undenatured Type II Collagen Singly or in Combination with Glucosamine and Chondroitin in Arthritic Dogs. Toxicol. Mech. Methods.

[16] Yora The Most Sustainable Dog Food in the World, Made in the UK. https://www.yorapetfoods.com/yora-pet-foods.

[17] Bhathal A., Spryszak M., Louizos C., Frankel G. (2017). Glucosamine and Chondroitin Use in Canines for Osteoarthritis: A Review. Open Vet. J..

[18] Dodge G.R., Regatte R.R., Noyszewski E.A., Hall J.O., Sharma A.V., Callaway D.A., Reddy R. (2011). The Fate of Oral Glucosamine Traced by 13C Labeling in the Dog. Cartilage..

[19] Martel-Pelletier J. (1999). Pathophysiology of Osteoarthritis. Osteoarthr. Cartil..

[20] Martel-Pelletier J., Tardif G., Fernandes J., Pelletier J.-P., Tsokos G.C. (2000). Metalloproteases and Their Modulation as Treatment in Osteoarthritis. Principles of Molecular Rheumatology.

[21] Rawlings N.D., Barrett A.J. (1994). Families of serine peptidases. Methods in Enzymology.

[22] Dozier W.A., Hess J.B. (2011). Soybean Meal Quality and Analytical Techniques. Soybean Nutr..

[23] Hanada M., Takahashi M., Furuhashi H., Koyama H., Matsuyama Y. (2016). Elevated Erythrocyte Sedimentation Rate and High-Sensitivity C-Reactive Protein in Osteoarthritis of the Knee: Relationship with Clinical Findings and Radiographic Severity. Ann. Clin. Biochem..

[24] Bao N., Zhou L., Cong Y., Guo T., Fan W., Chang Z., Zhao J. (2013). Free Fatty Acids Are Responsible for the Hidden Blood Loss in Total Hip and Knee Arthroplasty. Med. Hypotheses.

[25] Sogi Y., Yabe Y., Hagiwara Y., Tsuchiya M., Onoda Y., Sekiguchi T., Itaya N., Yoshida S., Yano T., Suzuki K. (2020). Joint Hemorrhage Accelerates Cartilage Degeneration in a Rat Immobilized Knee Model. BMC Musculoskelet. Disord..

[26] Fuentes-Lemus E., Dorta E., Escobar E., Aspée A., Pino E., Abasq M.L., Speisky H., Silva E., Lissi E., Davies M.J. (2016). Oxidation of Free, Peptide and Protein Tryptophan Residues Mediated by AAPH-Derived Free Radicals: Role of Alkoxyl and Peroxyl Radicals. RSC Adv..

[27] Karimi G., Hassanzadeh M., Mehri S. (2005). Protective Effect of *Rosmarinus Officinalis,* L. Essential Oil against Free Radical-Induced Erythrocyte Lysis. Iran. J. Pharm. Sci..

[28] López-Alarcón C., Rocco C., Lissi E., Carrasco C., Squella J.A., Nuñez-Vergara L., Speisky H. (2005). Reaction of 5-Aminosalicylic Acid with Peroxyl Radicals: Protection and Recovery by Ascorbic Acid and Amino Acids. Pharm. Res..

[29] van Dalen S.C.M., Kruisbergen N.N.L., Walgreen B., Helsen M.M.A., Slöetjes A.W., Cremers N.A.J., Koenders M.I., van de Loo F.A.J., Roth J., Vogl T. (2018). The Role of NOX2-Derived Reactive Oxygen Species in Collagenase-Induced Osteoarthritis. Osteoarthr. Cartil..

[30] Padgett L.E., Broniowska K.A., Hansen P.A., Corbett J.A., Tse H.M. (2013). The Role of Reactive Oxygen Species and Proinflammatory Cytokines in Type 1 Diabetes Pathogenesis. Ann. N. Y. Acad. Sci..

[31] Chakrabarti S., Jahandideh F., Wu J. (2014). Food-Derived Bioactive Peptides on Inflammation and Oxidative Stress. BioMed Res. Int..

[32] Boly R., Franck T., Kohnen S., Lompo M., Guissou I.P., Dubois J., Serteyn D., Mouithys-Mickalad A. (2015). Evaluation of Antiradical and Anti-Inflammatory Activities of Ethyl Acetate and Butanolic Subfractions of *Agelanthus Dodoneifolius* (DC.) Polhill & Wiens (Loranthaceae) Using Equine Myeloperoxidase and Both PMA-Activated Neutrophils and HL-60 Cells. Evid. Based Complement. Altern. Med. ECAM.

[33] Eurofins Food Testing NL. https://www.eurofins.com/contact-us/worldwide-interactive-map/the-netherlands/eurofins-food-testing-nl/.

[34] Murugesan S., Venkateswaran M.R., Jayabal S., Periyasamy S. (2020). Evaluation of the Antioxidant and Anti-Arthritic Potential of *Zingiber Officinale* Rosc. by in Vitro and in Silico Analysis. S. Afr. J. Bot..

[35] Ielciu I., Mouithys-Mickalad A., Franck T., Angenot L., Ledoux A., Păltinean R., Cieckiewicz E., Etienne D., Tits M., Crişan G. (2019). Flavonoid Composition, Cellular Antioxidant Activity and (Myelo)Peroxidase Inhibition of a *Bryonia Alba,* L. (Cucurbitaceae) Leaves Extract. J. Pharm. Pharmacol..

